# Development and Mechanistic Studies of Ternary Nanocomposites for Hydrogen Production from Water Splitting to Yield Sustainable/Green Energy and Environmental Remediation

**DOI:** 10.3390/polym14071290

**Published:** 2022-03-23

**Authors:** Asim Jilani, Syed Zajif Hussain, Ammar A. Melaibari, Nidal H. Abu-Hamdeh

**Affiliations:** 1 Center of Nanotechnology, King Abdulaziz University, Jeddah 21589, Saudi Arabia; aamelaibari@kau.edu.sa; 2 Department of Chemistry & Chemical Engineering, SBA-School of Science & Engineering (SBA-SSE), Lahore University of Management Sciences (LUMS), Lahore 54792, Pakistan; syed.hussain@lums.edu.pk; 3 Department of Mechanical Engineering, King Abdulaziz University, Jeddah 21589, Saudi Arabia; nabuhamdeh@kau.edu.sa; 4 Center of Research Excellence in Renewable Energy and Power System, King Abdulaziz University, Jeddah 21589, Saudi Arabia

**Keywords:** water treatment, photocatalytic hydrogen production, nanocomposites, antibiotic, photocatalytic degradation

## Abstract

Photocatalysts lead vitally to water purifications and decarbonise environment each by wastewater treatment and hydrogen (H_2_) production as a renewable energy source from water-photolysis. This work deals with the photocatalytic degradation of ciprofloxacin (CIP) and H_2_ production by novel silver-nanoparticle (AgNPs) based ternary-nanocomposites of thiolated reduce-graphene oxide graphitic carbon nitride (AgNPs-S-rGO_2%_@*g*-C_3_N_4_) material. Herein, the optimised balanced ratio of thiolated reduce-graphene oxide in prepared ternary-nanocomposites played matchlessly to enhance activity by increasing the charge carriers’ movements via slowing down charge-recombination ratios. Reduced graphene oxide (rGO), >2 wt.% or <2 wt.%, rendered H_2_ production by light-shielding effect. As a result, CIP degradation was enhanced to 95.90% by AgNPs-S-rGO_2%_@*g*-C_3_N_4_ under the optimised pH(6) and catalyst dosage(25 mg/L) irradiating beneath visible-light (450 nm, 150 watts) for 70 min. The chemical and morphological analysis of AgNPs-S-rGO_2%_@*g*-C_3_N_4_ surface also supported the possible role of thiolation for this enhancement, assisted by surface plasmon resonance of AgNPs having size < 10 nm. Therefore, AgNPs-S-rGO_2%_@*g*-C_3_N_4_ has 3772.5 μmolg^−1^ h^−1^ H_2_ production, which is 6.43-fold higher than *g*-C_3_N_4_ having cyclic stability of 96% even after four consecutive cycles. The proposed mechanism for AgNPs-S-rGO_2%_@*g*-C_3_N_4_ revealed that the photo-excited electrons in the conduction-band of *g*-C_3_N_4_ react with the adhered water moieties to generate H_2_.

## 1. Introduction

Hydrogen (H_2_) production through the photolysis of water is an alternative renewable green energy source with the prime advantages of harvesting energy and eco-friendly nature. The splitting of water under solar radiation or a light source of suitable wavelength is pertinent to the advent of new photocatalysts [1,2]. For this, various nanostructured semiconducting materials have been explored, such as titanium dioxide (TiO_2_), (*g*-C_3_N_4_), cadmium sulphide (CdS), zinc oxide (ZnO) and cerium(IV) [3]. However, the efficiency of single semiconductor photocatalysts is low due to the absorption of light in a small range of spectrum and the poor charge separation abilities leading to a high rate of electron-hole pair recombination. These problems have been addressed by engineering the appropriate bandgap and improving charge-recombination ratios or electron affinities for a radiation source using a combination of semiconducting materials with conducting oxides of metals or non-metals [4,5]. This combination develops the heterojunctions at recombinants’ interfaces and results in band-bending to charge separations by forcing their photogenerated charge carriers to move in reverse directions [6,7]. Therefore, by using the combination of binary or tertiary nanocomposite-based photocatalysts, the maximum H_2_ production can be obtained from water under certain experimental conditions by overcoming the listed issues [8].

Ciprofloxacin (CIP) is a broad-spectrum antibiotic with low-biodegradability being discharged at high volumes ranging from μg/L to mg/L into wastewaters of hospitals and pharmaceutical industries [9]. Its rise into water bodies poses a significant threat to the environment by increasing drug resistance in bacteria. Its elimination from the environment thus requires an urgent and efficient means. Accordingly, different techniques such as physical adsorption onto various substrates and biological, electrochemical, and photocatalytic degradations have been reported [10]. Among these, the photocatalytic approach has been most distinguished in eliminating the CIP pollutants due to high effectivity and ecological sustainability. The charge-carriers such as electron and hole (e^−^and h^+^) pairs produced by these photocatalysts, all under the influence of light-irradiation, are the source to generate the reactive-oxygen spices such as hydroxyl and superoxide (^·^OH and O₂⁻) radicals, responsible for the degradation of CIP molecules. It has been observed that the degradation of CIP molecules has been enhanced using heterojunctions or composites of semiconducting materials with metal nanoparticles [11]. The doping of sulphur (S) in *g*-C_3_N_4_ and rGO helped us modify their electrical conductivity and bandgap required for photodegradation of CIP molecules. This doping of S into these semiconductors further supports forming the smaller sized AgNPs. It results from changes in their pristine optical absorptions by activating these photocatalysts under higher visible ranges.

Among the previously mentioned photocatalysts, *g*-C_3_N_4_ has attained the scientific community’s attention for photocatalytic H_2_ production. The popularity of *g*-C_3_N_4_ is attributed to its non-toxicity, ease of synthesis, and tuneable nature of its bandgap [12]. Furthermore, its physio-chemical stability is due to covalent bonding between nitrogen and carbon, allowing it to function equally well in acidic and basic media. Additionally, the negative band potential of *g*-C_3_N_4_ (around 1.3 eV) facilitates photocatalytic H_2_ production and water oxidation reaction under visible light [13]. However, the competence of pure *g*-C_3_N_4_ for photocatalytic H_2_ production is far less due to rapid charge-recombination ratios and the existence of a wide bandgap (of around 2.7 eV). Therefore, doping of *g*-C_3_N_4_ with other materials such as cadmium sulphide, molybdenum disulphide, gold, graphene and low dimension carbon-based materials supports the suppression of rapid charge recombinations and narrows the bandgap, which is highly required for large-scale photocatalytic H_2_ production [14,15]. Among these materials, reduced graphene oxide (rGO) seems a promising co-catalyst due to its conjugated structure, high specific surface area, and the tuneable nature of electronic bands [16].

Notably, the proportion of rGO as co-catalyst in the composite catalyst is crucial for photocatalytic H_2_ production and energy application [17]. The higher amount of rGO produces negative shielding effects [18], which slow down the charge carriers’ movement from the valence band (VB) to the conduction band (CB), ultimately affecting the H_2_ production. Consequently, the efficiency of rGO@*g*-C_3_N_4_ can be further enhanced after adjusting the amount of rGO in *g*-C_3_N_4_ to a specific ratio. Additionally, doping with elements with low electronegativities, such as boron (B) and sulphur (S), can also boost the catalytic efficiency by raising the Fermi level [19]. Thus, the thiolation of composite materials, rGO and *g*-C_3_N_4_, can enhance photocatalytic H_2_ production in two ways, by dropping the charge-recombination ratios and increasing the light absorption adeptness. The thiolation help to conquer the negative shielding effect of thiolated rGO (HS-rGO), increasing its absorption efficiency and the increased carbon vacancies in thiolated *g*-C_3_N_4_ (HS-*g*-C_3_N_4_) act as active territories to reduce the chance of charge recombinations [20]. In summary, thiolation increases the charge separation capabilities of both HS-rGO and HS-*g*-C_3_N_4_. Interestingly, photocatalytic H_2_ production can be further improved by coupling thiolated composites with some noble metal nanoparticles (NPs), such as silver. The silver nanoparticles (AgNPs) are highly conductive and support photocatalysis for H_2_ production through surface plasmon resonance (SPR). This SPR property also assists the movement of charge carriers, enhancing photocatalytic H_2_ production [21,22].

A literature review showed that various groups had synthesised rGO and its composites with *g*-C_3_N_4_ at a fixed amount of rGO for photocatalytic hydrogen production. In this context, Jaiswal et al. prepared the rGO-based composites of *g*-C_3_N_4_ using a constant 5 wt.% [23]. Likewise, Ibrahim and his colleagues investigated the TiO_2_/rGO/*g*-C_3_N_4_ system for H_2_ production with a fixed 1 wt.% of rGO [24]. In the same way, Pan et al. [25] explored the effect of 0.3% rGO in their rGO@*g*-C_3_N_4_ composites for hydrogen production. However, the impact of thiolation has not been analysed, and the concentration of thiolated rGO (HS-rGO) in ternary-nanocomposites for H_2_ production can be further optimised.

In view of the previous reports [12,19,20,23,24,25], rGO-based composites of *g*-C_3_N_4_ were prepared at different concentrations of HS-rGO, from 1 to 4 wt.%. The thiolated composite (HS-rGO@*g*-C_3_N_4_) with an optimised concentration of HS-rGO of 2 wt.% was selected based on photoluminescence (PL) studies which indicated lesser charge recombinations. HS-rGO_2%_@*g*-C_3_N_4_ was found to have improved structural and chemical properties and is highly desirable for photocatalytic H_2_ production. Next, the HS-rGO_2%_@*g*-C_3_N_4_ was decorated with AgNPs to produce AgNPs-S-rGO_2%_@*g*-C_3_N_4_. The charge transfer, structural, surface chemical, optical conduction, and photocatalytic H_2_ production properties of this final nanocomposite, AgNPs-S-rGO_2%_@*g*-C_3_N_4_, were explored under the same experimental conditions compared to HS-rGO_2%_@*g*-C_3_N_4_ and rGO_2%_@*g*-C_3_N_4_.

## 2. Experimental Details

### 2.1. Synthesis and Thiolation of rGO

Sheets of graphene oxide (GO) were synthesised using a modified Hummer method as per our previous work [26]. After this, GO sheets were heated to 200 °C for 2 h to obtain rGO. This rGO was thiolated following an earlier report with minor modifications [27]: 50 mg of rGO was dispersed in 50 mL of ultrapure deionised-water and ultrasonicated for 1.5 h, followed by the addition of 3 mL of HBr and further sonication for 2 h. After this, 2.5 g of thiocarbamide was added at 80 °C and stirred continuously for 24 h. The reaction was stopped, and the reaction media was allowed to cool to room temperature. Afterwards, 25 mL of 4 M aqueous solution of KOH was added and stirred for a further 1 h. The black suspension of thiolated GO (HS-rGO) was filtrated through a PTFE membrane filter (0.2 µm) and washed many times with dimethyl ether, ethanol, and water. The resulting HS-rGO nanosheets were redispersed into ultrapure water by sonication and vacuum dried before further use.

### 2.2. Synthesis of Graphitic Carbon Nitride Nanosheets (*g*-C_3_N_4_)

To prepare *g*-C_3_N_4_, a pale-yellow powder, 2.5 g of melamine was heated in the muffle-furnace to 525 °C by providing 10 °C min^−1^ heating rate as per previous reports by Zhang et al. [28].

### 2.3. Synthesis of Thiolated Graphitic Carbon Nitride Nanosheets (HS-*g*-C_3_N_4_)

HS-*g*-C_3_N_4_ was prepared by following the Wang’s protocol via the single-step thermal-polymerisation of thiocarbamide [13]. For this, 30 g of thiocarbamide was kept in a porcelain crucible with no lid. This open crucible was placed into another large crucible bolted with a lid and placed to heat in a muffle-furnace to 550 °C for 5 h by providing a temperate rate of 2 °C min^−1^. This process resulted in a pale-yellow coloured powder, HS-*g*-C_3_N_4_, which accumulated in the larger crucible.

### 2.4. Fabrication of rGO@*g*-C_3_N_4_ and HS-rGO@*g*-C_3_N_4_

To fabricate rGO@*g*-C_3_N_4_ with different concentrations of rGO, 4.7 g of *g*-C_3_N_4_ was added separately into 15 mL solution of rGO prepared using 1, 2, 3 and 4 wt.% rGO according to the measured mass of *g*-C_3_N_4_. These four mixtures were then vigorously sonicated under slow stirring for 1 h to coat rGO onto the *g*-C_3_N_4_. Next, the rGO-coated *g*-C_3_N_4_ (rGO@*g*-C_3_N_4_) was filtered from the solution and vacuum dried. The rGO@*g*-C_3_N_4_ with 2 wt.% of rGO (rGO_2%_@*g*-C_3_N_4_) was found best, as illustrated by the PL study. Further, the same composite was prepared using 2 wt.% of HS-rGO to HS-*g*-C_3_N_4_ (HS- rGO_2%_@*g*-C_3_N_4_). Finally, the HS- rGO_2%_@*g*-C_3_N_4_ was decorated with AgNPs to make the ternary composites.

### 2.5. Synthesis of AgNPs onto the Surface of HS-rGO_2%_@*g*-C_3_N_4_

**Figure 1:**
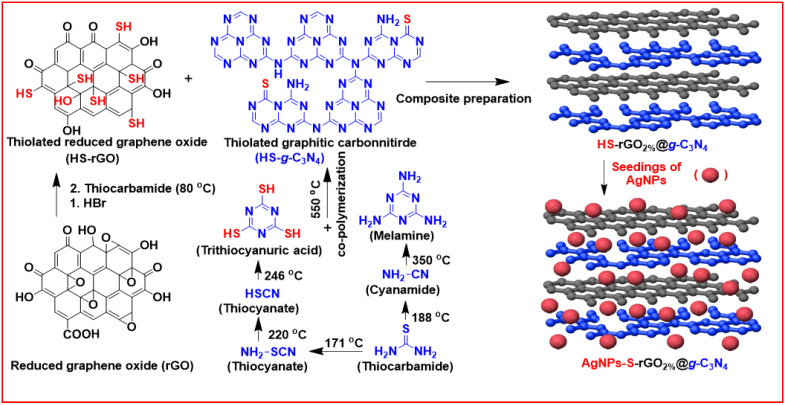
Scheme for the synthesis of AgNPs-S-rGO_2%_@*g*-C_3_N_4_.

AgNPs were prepared over HS-rGO_2%_@*g*-C_3_N_4_ nanosheets via the reduction of silver ions. In this context, 45 mg of HS-rGO_2%_@*g*-C_3_N_4_ nanosheets were mixed into 50 mL (2 mM) of AgNO_3_ solution in a round bottom flask fixed in an ice bath under continuous stirring. Under these conditions, this mixture was sonicated for 30 min to obtain a uniform distribution. Then 20 mL (25 mM) of NaBH_4_ solution was added dropwise to reduce the silver ions in the prepared dispersion to prepare the AgNPs and attach them to the thiol positions on the nanosheets to yield AgNP-decorated HS-rGO_2%_@*g*-C_3_N_4_ (AgNPs-S-rGO_2%_@*g*-C_3_N_4_**)**. Finally, the prepared AgNPs-S-rGO_2%_@*g*-C_3_N_4_ nanosheets were washed several times with deionised water and methanol by centrifuging them at 8000 rpm, and vacuum dried before further use. The synthetic scheme of AgNPs-S-rGO_2%_@*g*-C_3_N_4_ (Figure 1).

### 2.6. Characterisations

Structural properties of *g*-C_3_N_4_, HS-rGO@*g*-C_3_N_4_ and AgNPs-S-rGO@*g*-C_3_N_4_ were studied between diffraction angles from 5 to 80° using X-ray diffraction (XRD), Rigaku Ultima-IV Tokyo Japan. The optical properties were explored using photoluminescence spectroscopy (PL-Kimon 1.K) and UV-Visible diffuse reflectance spectroscopy (HACH LANGE DR 6000). Surface and functional analysis were performed by X-ray photoelectron spectroscopy (PHI-VersaProbII, PHI, Chanhassen, MN, USA) under a high vacuum (10^−7^ Pa). Morphology and chemical nature of these samples were explored using a Nova NanoSEM 450 (FEI, Hillsboro, OR, USA) equipped with an Everhart-Thornley detector (ETD), a scanning transmission electron detector (STEM), and an energy dispersive X-ray detector (EDX), operated between 15 to 25 kV at a working distance of 5 mm. The evolution of H_2_ was scrutinised using gas chromatography (Agilent Technologies 7890B-GC System, Santa Clara, CA, USA).

### 2.7. Hydrogen Production

The photocatalytic study was conducted in a Pyrex beaker that contained 15 mg of AgNPs-S-rGO_2%_@*g*-C_3_N_4_. The same sacrificial reagent (15% triethanolamine) was used in all experiments. However, before starting the photocatalytic generation of H_2_, surface-adsorbed oxygen moieties were removed by degassing this reaction assembly with sustained nitrogen gas flow for 1 h. These experiments have been performed under visible light (150 Watt, Xe lamp). The evolution of H_2_ was scrutinised at regular intervals of 30 min, using gas chromatography (Agilent Technologies, 7890B-GC System). Furthermore, AgNPs-S-rGO_2%_@*g*-C_3_N_4_ was tested for reusability under the same experimental conditions by four times repeating the experiment.

### 2.8. Photocatalytic Degradation of Ciprofloxacin Antibiotics

A Ciprofloxacin antibiotic (CIP) was chosen as a model pollutant for the photocatalytic ability of AgNPs-S-rGO_2%_@*g*-C_3_N_4_. Therefore, 25 mg/L concentration of CIP was prepared. The photocatalytic degradation of CIP was investigated under visible light (450 nm, 150 watts, distance from light source to CIP solution 40 cm, and light intensity 74.64 w/m^2^) at room temperature while maintaining pH 7 during the study. However, the solution was kept in the dark for 20 min before irradiating to achieve the absorption equilibrium. Afterwards, the CIP solution was started irradiating, and 3 mL was taken with the regular interval of 10 min to analyse the degradation of CIP. The following equation was used to calculate the degradation parameters of CIP [29].

(1) $$\mathrm{Degradation}\;\left(\%\right)=\left(\frac{C_{o}-C_{\;}}{C_{o}}\;\right)100$$

In the above equation, $C_{o}$ and $C_{\;}\;$ represents the initial and final concentration of CIP, respectively. Further, the following equation was used to calculate the kinetic rate constant (*k*) during photocatalytic degradation of CIP [30].

(2) $$\ln\left(\frac{C}{C_{o}}\right)=-kt$$

## 3. Results and Discussion

### 3.1. Structural Analysis

**Figure 2:**
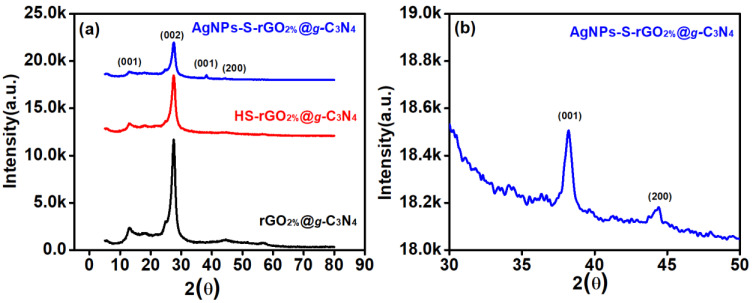
(**a**) Diffraction patterns of rGO_2%_@*g*-C_3_N_4_, HS-rGO_2%_@*g*-C_3_N_4_ and AgNPs-S-rGO_2%_@*g*-C_3_N_4_ whilst (**b**) enlarged spectra of AgNPs-S-rGO_2%_@*g*-C_3_N_4_ taken between 30 to 50°.

The diffraction pattern of rGO_2%_@*g*-C_3_N_4_ shown in Figure 2 indicated that the peaks that appear at around 2θ = 13.26° and 27.46° are attributed to the (001) and (002) diffraction planes, respectively, of the hexagonal form of *g*-C_3_N_4_ (JCPDS # 01-087-1526) [31]. The diffraction plane (001) is associated with in-plane structural packing of tri-s-triazine with an interlayer spacing of 0.66 nm.

In comparison, (002) is related to interlayer aromatic stacking of C-N having the interlayer spacing of 0.32 nm [32]. However, the diffraction peaks of rGO could not be seen due to the reduction in functional moieties anchored with its basal plane. The absence of diffraction peaks for rGO is consistent with an earlier study [33]. Compared to rGO_2%_@*g*-C_3_N_4_, the peak (001) in the diffraction patterns of HS-rGO_2%_@*g*-C_3_N_4_ was shifted from 2θ = 13.26° to 12.99°, whereas (002) moved from 2θ = 27.46° to 27.5°. After thiolation, the shifting of the diffraction planes is attributed to incorporating the sulphur atoms into the copolymerised sheets of the *g*-C_3_N_4_ and within the layered assemblies of the rGO. This incorporation resulted in the reduction of the thickness of the layers in these composites [13]. In the case of AgNPs-S-rGO_2%_@*g*-C_3_N_4_, additional diffraction peaks at around 38.18° and 44.36° were observed. These were, respectively, attributed to the (111) and (200) planes of silver, as per JCPDS # 00-001-1164. Moreover, in AgNPs-S-rGO_2%_@*g*-C_3_N_4_, the small shifts of the (001) and (002) peaks towards lower diffraction angles are attributed to the successful interaction of AgNPs with HS-rGO_2%_@*g*-C_3_N_4_. These shifts are associated with the covalent interactions of AgNPs with the thiol moieties of HS-rGO_2%_@*g*-C_3_N_4_, which separates and keeps intact the layered assemblies of the composite material. This is due to the difference in atomic radii of silver, carbon and nitrogen [34]. Ag has a higher atomic radius (0.144 nm) in comparison to carbon (0.077 nm) and nitrogen (0.075 nm) [34]. Therefore, this difference was valuable for promoting successful interaction, which further enhanced light harvesting, resulting in higher photocatalytic H_2_ production.

### 3.2. Optical Properties

**Figure 3:**
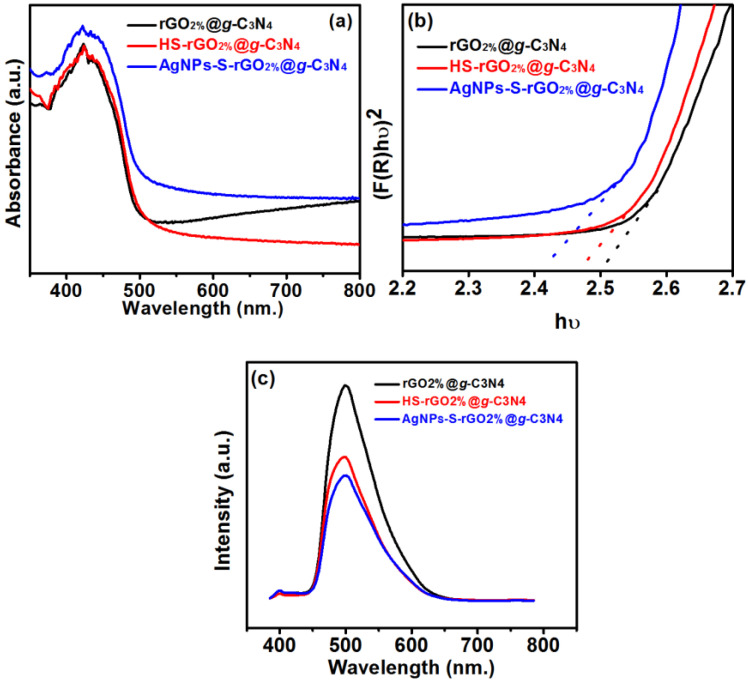
(**a**) UV-Visible absorption spectra (**b**) bandgap analysis and (**c**) PL spectra for rGO_2%_@*g*-C_3_N_4_, HS-rGO_2%_@*g*-C_3_N_4_, and AgNPs-S-rGO_2%_@*g*-C_3_N_4_.

The optical properties give insight into the charge transfer from the VB to the CB, directly impacting the material’s ability to generate H_2_ from the water splitting. Therefore, UV-Visible diffuse reflectance spectroscopy was employed to explore the charge transfer conduction rate of rGO_2%_@*g*-C_3_N_4_, HS-rGO_2%_@*g*-C_3_N_4_, and AgNPs-S-rGO_2%_@*g*-C_3_N_4_. The absorption peaks of rGO_2%_@*g*-C_3_N_4_ were shifted to a higher wavelength after thiolation (HS-rGO_2%_@*g*-C_3_N_4_) and moved further following seeding with AgNPs (AgNPs-S-rGO_2%_@*g*-C_3_N_4_), as shown in Figure 3a. Compared to rGO_2%_@*g*-C_3_N_4_, an increase in the light absorption ability of HS-rGO_2%_@*g*-C_3_N_4_ is attributed to thiolation [35]. The associated thiol moieties are excellent hole quenchers and are well known to reduce the recombination rates of electron-hole pairs in the resulting composites [20]. Further, in the case of AgNPs-S-rGO_2%_@*g*-C_3_N_4_, the characteristic SPR of AgNPs enhances light absorption. However, the SPR is dependent on the size of the AgNPs. However, a non-observable SPR peak hump was noticed for AgNPs-S-rGO_2%_@*g*-C_3_N_4_ caused by two possible reasons (Figure 3a). First, the interaction of thiolated rGO with AgNPs can lead to quenching the SPR in AgNPs-S-rGO_2%_@*g*-C_3_N_4_ because of the difference in their dielectric constants [35]. A second possible reason may be low concentrations or average particle size (<10 nm). Thus, if the AgNPs have a size within a range of a few nanometres or the amount impregnated is low, their SPR will remain imperceptible [36]. In our case, the size of the AgNPs that could be seen in TEM analysis was found to be less than 10 nm, but their low concentration as determined by the XPS survey scan (Figure S2 and Table S1, Supplementary Materials) could be the second reason for the absence of the AgNPs SPR in the absorbance spectrum of AgNPs-S-rGO_2%_@*g*-C_3_N_4_.

The change in the absorbance of light can further transform the conduction behaviour of the subjected material, which negatively affects hydrogen production. Therefore, the Kubelka–Munk function was used to estimate the conduction behaviour of rGO_2%_@*g*-C_3_N_4_, HS-rGO_2%_@*g*-C_3_N_4_ and AgNPs-S-rGO_2%_@*g*-C_3_N_4_ (Figure 3b) [37]. The bandgap calculated for rGO_2%_@*g*-C_3_N_4_ was 2.50 eV, which is less than that of *g*-C_3_N_4_ [38]. Further, reduction in bandgap value of *g*-C_3_N_4_ after the addition of rGO is ascribed to the interactions by covalent bonding between carbon atoms in *g*-C_3_N_4_ and rGO [39]. This interaction leads to the reduction of the bandgap for rGO_2%_@*g*-C_3_N_4_. The bandgap of rGO_2%_@*g*-C_3_N_4_ was further reduced after thiolation and was about 2.46 eV for HS-rGO_2%_@*g*-C_3_N_4_. This decrease in the bandgap after thiolation is attributed to the enhanced strong affinity between the functional groups of *g*-C_3_N_4_ and rGO, leading to improved absorption of light with enhanced charge transfer behaviour between their CB the VB [40]. Further, the bandgap was reduced to 2.42 eV AgNPs-S-rGO_2%_@*g*-C_3_N_4_. However, during this transfer, these charge carriers may recombine, which hinders H_2_ production. Therefore, the charge recombination ratio of rGO_2%_@*g*-C_3_N_4_, HS-rGO_2%_@*g*-C_3_N_4_, and AgNPs-S-rGO_2%_@*g*-C_3_N_4_ was estimated using the PL spectra (Figure 3c). The intensity of the PL spectra is directly proportional to the ratio of charge recombinations. The PL spectra of pure *g*-C_3_N_4_ (Figure S1) showed strong emission, attributed to the rapid recombinations of charge carriers. However, the PL intensity was reduced with the addition of rGO to the *g*-C_3_N_4_. The addition of rGO to *g*-C_3_N_4_ behave as shuttle for excited electrons to move from *g*-C_3_N_4_ to rGO at the interface between them and it deters the charge recombination [41]. Therefore, the intensity of the PL spectra for rGO_1%_@*g*-C_3_N_4_ (Figure S1) was less than that of the pristine *g*-C_3_N_4_ and was further reduced for rGO_2%_@*g*-C_3_N_4_. However, as the percentage of rGO increased from 2 wt.% to 3 wt.% and 4 wt.% (Figure S1), the PL intensity increased, which indicates the rapid recombination of charged particles in rGO_3%_@*g*-C_3_N_4_ and rGO_4%_@*g*-C_3_N_4_. The increase in PL intensity at a higher percentage (above 2% in our case) is attributed to the negative shielding effect of rGO, which enhanced the charge-recombination ratios for rGO_3%_@*g*-C_3_N_4_ and rGO_4%_@*g*-C_3_N_4_ [42]. Therefore, we selected rGO_2%_@*g*-C_3_N_4_ for thiolation. The PL intensity of HS-rGO_2%_@*g*-C_3_N_4_ was reduced than that of rGO_2%_@*g*-C_3_N_4_ and was further reduced (Figure 3c) after adding AgNPs. The reduction of the PL intensity after thiolation and seeding with AgNPs (AgNPs-S-rGO_2%_@*g*-C_3_N_4_) showed that the charge recombination ratio has reduced to the point desired for enhanced H_2_ production.

### 3.3. Surface Chemical State Investigation

**Figure 4:**
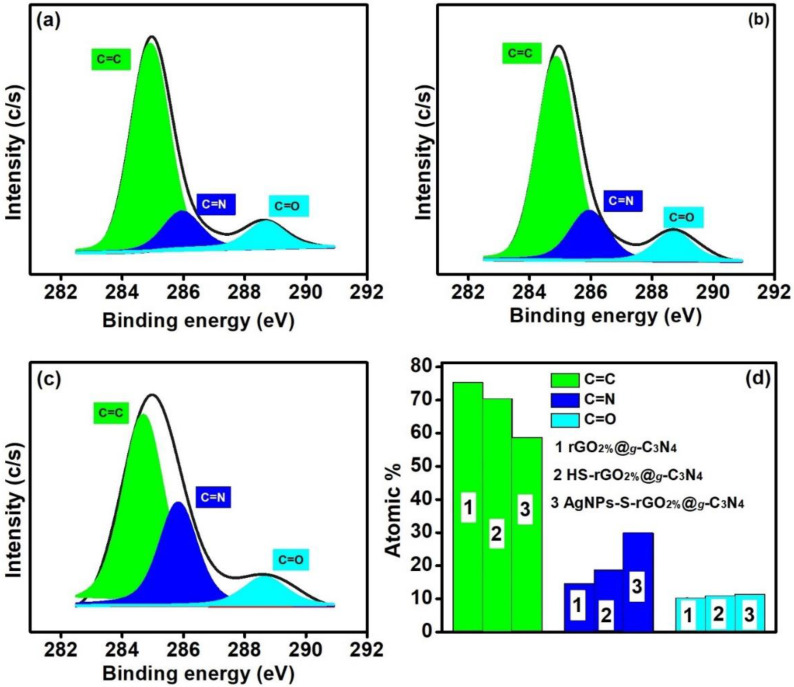
Chemical state analysis of C1s (**a**) rGO_2%_@*g*-C_3_N_4_, (**b**) HS-rGO_2%_@*g*-C_3_N_4_, (**c**) AgNPs-S-rGO_2%_@*g*-C_3_N_4_ and (**d**) apparent change in functional groups for rGO_2%_@*g*-C_3_N_4_, HS-rGO_2%_@*g*-C_3_N_4,_ and AgNPs-S-rGO_2%_@*g*-C_3_N_4_.

The C1s spectrum of rGO_2%_@*g*-C_3_N_4_ (Figure 4a) revealed three peaks at around 284.9, 285.8, and 288.6 eV accredited to the C=C, C=N, and C=O moieties, respectively [43,44], with contributions of 75.28%, 14.55%, and 10.17%. However, in thiolated HS-rGO_2%_@*g*-C_3_N_4_ and AgNPs coated composites (AgNPs-S-rGO_2%_@*g*-C_3_N_4_) (Figure 4b,c), the bonding of detected elements was changed as reflected by changes in their spectra. The percentage contributions of C=C, C=N, and C=O changed to 70.38%, 18.69%, and 10.93%, respectively, in HS-rGO_2%_@*g*-C_3_N_4_, while for AgNPs-S-rGO_2%_@*g*-C_3_N_4_, they were 58.77%, 29.80% and 11.43%, respectively. The increase in C=O suggests that more anchoring sites are available to enhance catalytic activity [45]. Therefore, AgNPs-S-rGO_2%_@*g*-C_3_N_4_ also has a high photocatalytic H_2_ production rate in comparison to rGO_2%_@*g*-C_3_N_4_ and HS-rGO_2%_@*g*-C_3_N_4_ (Section 3.5). Further, Figure 4d shows the changes in C=C, C=N, and C=O of rGO_2%_@*g*-C_3_N_4_, HS-rGO_2%_@*g*-C_3_N_4,_ and AgNPs-S-rGO_2%_@*g*-C_3_N_4_.

**Figure 5:**
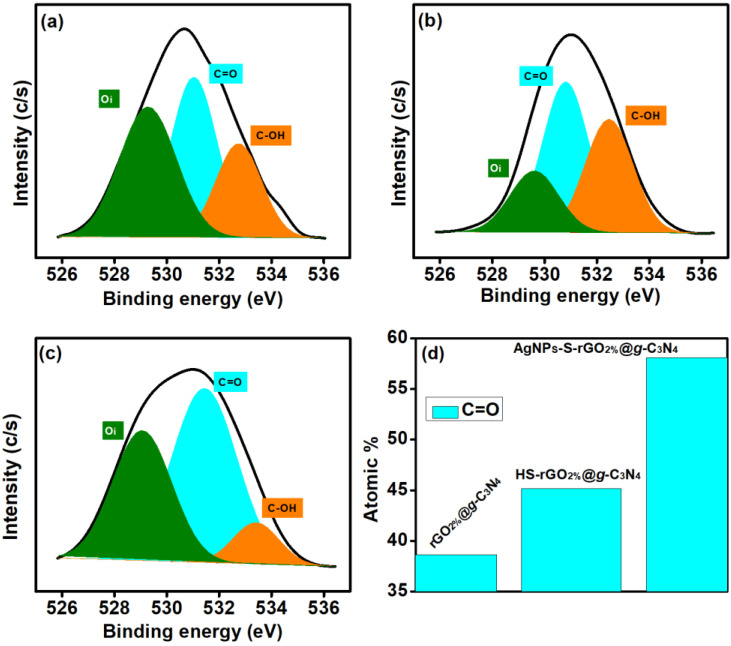
O1s-attached functional groups (**a**) rGO_2%_@*g*-C_3_N_4_, (**b**) HS-rGO_2%_@*g*-C_3_N_4_ (**c**) AgNP_S_-S-rGO_2%_@*g*-C_3_N_4_ and (**d**) increment in C=O bonding of rGO_2%_@*g*-C_3_N_4_, HS-rGO_2%_@*g*-C_3_N_4,_ and AgNP_S_-S-rGO_2%_@*g*-C_3_N_4_.

The O1s spectra of the prepared materials provided evidence of oxygen intercalations. The peaks that appeared at around 529.2, 531.0 and 532.3 eV are attributed to lattice oxygen (Oi), carbon to oxygen bonding (C=O), and hydroxyl functional groups (OH), respectively [46,47]. The rGO_2%_@*g*-C_3_N_4_ spectra showed the contribution of Oi to be around 38.82%, whereas the HS-rGO_2%_@*g*-C_3_N_4_ and AgNPs-S-rGO_2%_@*g*-C_3_N_4_ spectra contributed 19.49% and 36.78%, respectively (Figure 5a–c). The contribution of C=O was found to be maximised for AgNPs-S-rGO_2%_@*g*-C_3_N_4_ and was 54.11%, which is according to the spectrum of C1s (Figure 5d). The percentage of OH was around 22.55%, 35.34% and 9.12% for rGO_2%_@*g*-C_3_N_4_, HS-rGO_2%_@*g*-C_3_N_4_ and AgNPs-S-rGO_2%_@*g*-C_3_N_4_, respectively.

**Figure 6:**
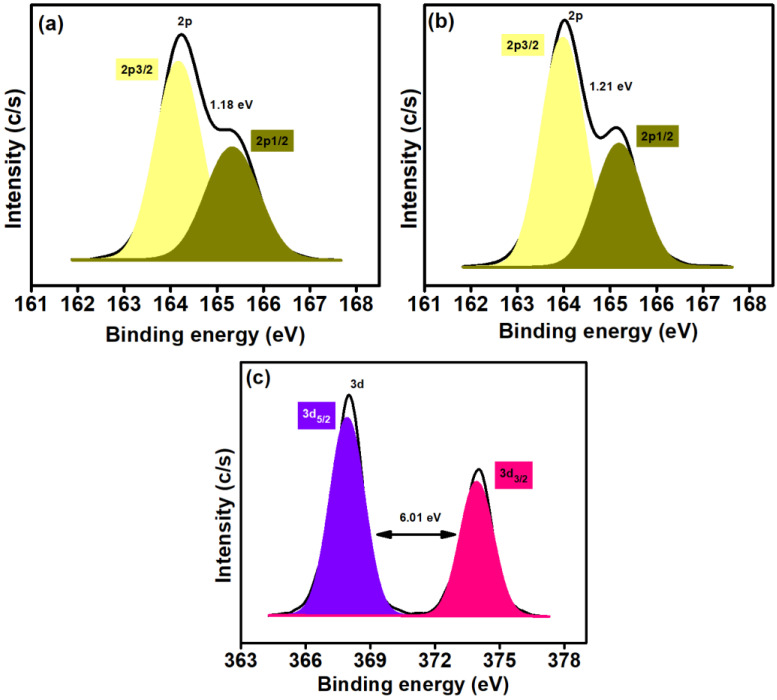
(**a**,**b**) S2p chemical state analysis of HS-rGO_2%_@*g*-C_3_N_4_ and AgNP_S_-S-rGO_2%_@*g*-C_3_N_4_ (**c**) Ag3d analysis for AgNPs-S-rGO_2%_@*g*-C_3_N_4_.

The S2p spectrum analysis of HS-rGO_2%_@*g*-C_3_N_4_ (Figure 6a) shows two peaks at around 164.17 and 165.33 eV, attributed to the 2p3/2 and 2p1/2, respectively, with a separation of 1.18 eV. However, after depositing the AgNPs on HS-rGO_2%_@*g*-C_3_N_4_, the 2p3/2 and 2p1/2 peaks were shifted to 163.98 and 165.19, increasing the separation of 1.21 eV (Figure 6b). This change in the binding position of the 2p3/2 and 2p1/2 peaks revealed the interaction of the AgNPs with the sulphur group of HS-rGO_2%_@*g*-C_3_N_4_.

Furthermore, the Ag3d spectrum of AgNPs-S-rGO_2%_@*g*-C_3_N_4_ (Figure 6c) was measured to explore the chemical nature of AgNPs. The peaks, which appeared at 367.93 and 373.93 eV, are attributed to Ag3d5/2 and Ag3d3/2, respectively, which confirms the existence of zerovalent silver [48]. Moreover, zerovalent silver in AgNPs is highly desirable as it enhances the rate of photocatalytic reactions. In the present work, this property of silver is also responsible for accelerating hydrogen production [49].

### 3.4. Surface Morphology

**Figure 7:**
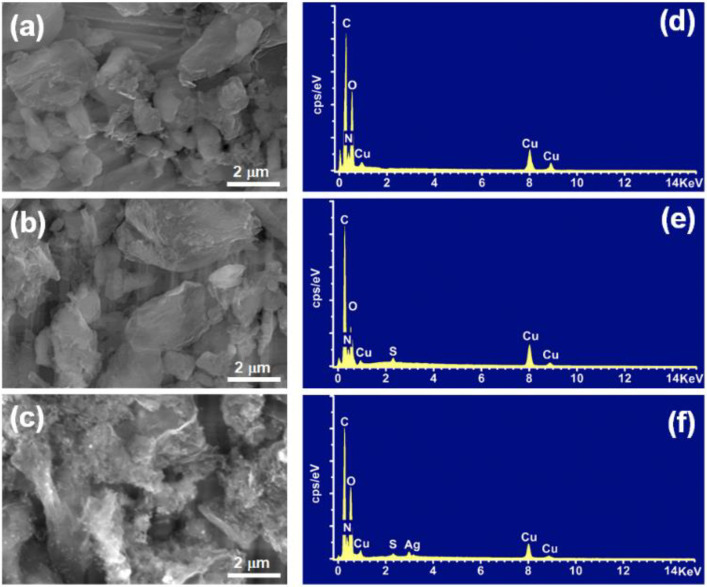
(**a**–**c**) SEM analysis and (**d**–**f**) EDS analysis of rGO_2_%@*g*-C_3_N_4_, HS-rGO_2_%@*g*-C_3_N_4_, and AgNPs-S-rGO_2_%@*g*-C_3_N_4_.

The well-dispersed aqueous droplets of rGO_2%_@*g*-C_3_N_4_, HS-rGO_2%_@*g*-C_3_N_4_, and AgNPs-S-rGO_2%_@*g*-C_3_N_4_ were drop-casted onto copper stubs and air-dried before scanning electron microscope (SEM) analysis was conducted. Likewise, for transmission electron microscope (TEM) analysis, the sample of AgNPs-S-rGO_2%_@*g*-C_3_N_4_ was prepared by drop-casting it onto a copper-grid followed by air-drying. The morphology appearing in the SEM images of rGO_2%_@*g*-C_3_N_4_, HS-rGO_2%_@*g*-C_3_N_4_, and AgNPs-S-rGO_2%_@*g*-C_3_N_4_ revealed that there is no interlayer stacking between *g*-C_3_N_4_ and rGO layers in their thiol-enriched (HS-rGO_2%_@*g*-C_3_N_4_) and AgNP-decorated (AgNPs-S-rGO_2%_@*g*-C_3_N_4_) composites compared to the non-functionalised (rGO_2%_@*g*-C_3_N_4_) composite (Figure 7a–c).

**Figure 8:**
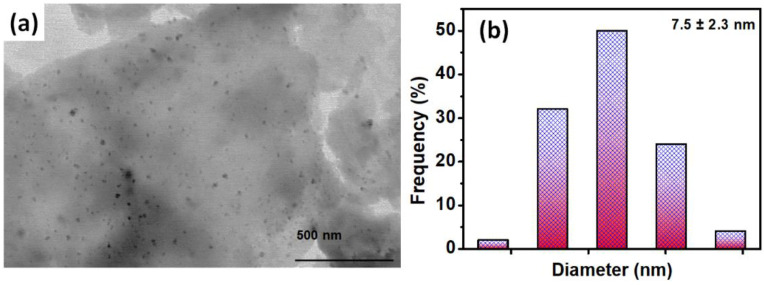
(**a**) Surface morphology of AgNPs-S-rGO_2%_@*g*-C_3_N_4_ via TEM and (**b**) the estimated size of the AgNPs was 7.5 ± 2.3 measured using imageJ software.

In parallel (Figure 7d–f), the thiol peaks indicated in the EDX spectrum have also confirmed the thiol enrichment in HS-rGO_2%_@g-C_3_N_4,_. According to XPS studies, the engagement of these thiol groups with NaBH_4_ reduced the silver ions under these experimental conditions, especially continuous sonication. It ensured the formation of AgNPs in the resulting AgNPs-S-rGO_2%_@*g*-C_3_N_4_ composite. Briefly, small-sized AgNPs (less than 10 nm) were formed between the interlayer stacking of *g*-C_3_N_4_ and rGO in AgNPs-S-rGO_2%_@g-C3N4, as seen in the TEM analysis shown in Figure 8.

### 3.5. Hydrogen Production and Proposed Mechanism for AgNPs-S-rGO_2%_@*g*-C_3_N_4_

**Figure 9:**
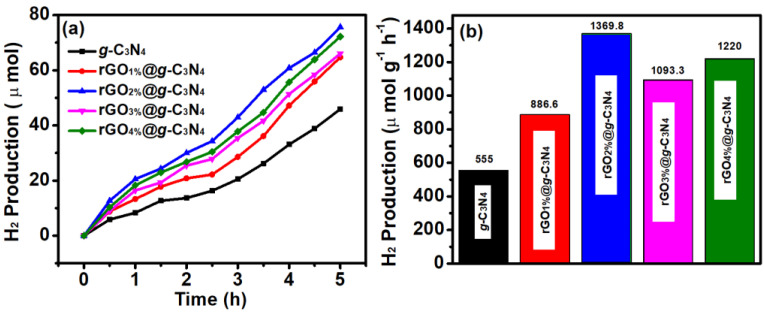
(**a**) H_2_ production and (**b**) H_2_ production rate per hour under the same experimental conditions for *g*-C_3_N_4_ and its composites with rGO at a concentration of 1 to 4 wt.%.

The photocatalytic H_2_ production by *g*-C_3_N_4_ (Figure 9a) had a production rate of 555-μmol g^−1^ h^−1^, and this low rate was accredited to the fast recombinations of charge carriers (electron-hole pair). This H_2_ production rate of *g*-C_3_N_4_ was further improved with the addition of rGO. In rGO_1%_@*g*-C_3_N_4_, the reaction rate increased to 886.66 μmol g^−1^ h^−1^, which is 1.59 fold of *g*-C_3_N_4_. This increment in rate exhibited the contribution of rGO to accelerating the charge transfer efficiencies by tumbling the speedy recombinations of charge carriers. The role of rGO in increasing the H_2_ production rate is consistent with the previous literature [50]. However, in these measurements, the H_2_ production rate was reached 1369.86 μmol g^−1^ h^−1^ with an increased weight percentage of rGO from 1% to 2% (rGO_2%_@*g*-C_3_N_4_), which is 2.4-fold higher than the rate of *g*-C_3_N_4_. This was the maximum H_2_ production rate achieved compared to 1, 3 and 4 wt.% loading of rGO and confirms the positive role of rGO as a co-catalyst in nanocomposites in enhancing catalytic performance. Thus, it is imperative to adjust the weight percentage of rGO in the subjected nanocomposites as its higher ratio can produce shielding effects, which ultimately reduces catalytic performance [51].

**Figure 10:**
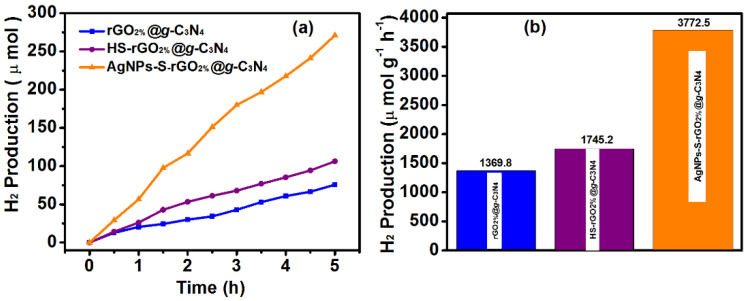
(**a**) Hydrogen production and (**b**) rate of hydrogen production per hour under the same set experimental conditions for rGO_2%_@*g*-C_3_N_4_, HS-rGO_2%_@*g*-C_3_N_4_ and AgNPs-S-rGO_2%_@*g*-C_3_N_4_.

Therefore, the catalytic performance was reduced by further increasing the weight percentage of rGO to 3% and 4%. The rGO_3%_@*g*-C_3_N_4_ showed H_2_ production rate of 1220 μmol g^−1^h^−1^, whilst rGO_4%_@*g*-C_3_N_4_ had a rate of 1093.33 μmol g^−1^h^−1^. This reduced hydrogen production above a specific weight percentage of added rGO is attributed to the light-shielding effect. This kind of result for a particular weight percentage of rGO has also been reported earlier [52]. Therefore, rGO_2%_@*g*-C_3_N_4_ was selected as the most optimised sample for maximum hydrogen production under set experimental conditions. Figure 9b shows the change in the H_2_ production rate with rGO to *g*-C_3_N_4_ at various concentrations of rGO. The introduction of thiol groups (HS) could further increase the H_2_ production rate of rGO_2%_@*g*-C_3_N_4_. In addition, the groups can act as hole quenchers which also reduce the charge recombination ratio [53]. Therefore, HS-rGO_2%_@*g*-C_3_N_4_ (after the thiolation of rGO_2%_@*g*-C_3_N_4_), Figure 10a shows the H_2_ production rate of 1745.23 μmol g^−1^h^−1^_,_ which was 1.27 times higher than the rate of rGO_2%_@*g*-C_3_N_4_. The HS groups have a strong affinity for noble transition metals; therefore, AgNPs were grown, using a chemical reduction method, over the surface of thiolated sheets of rGO_2%_@*g*-C_3_N_4_, resulting in AgNPs-S-rGO_2%_@*g*-C_3_N_4_. This AgNPs-S-rGO_2%_@*g*-C_3_N_4_ (Figure 10a) was found to generate hydrogen at a rate of 3772.5 μmol g^−1^h^−1^, which is 2.16- and 2.76-fold higher than HS-rGO_2%_@*g*-C_3_N_4_ and rGO_2%_@*g*-C_3_N_4_, respectively. Moreover, Table 1 shows the ternary composites is better than binary composites for photocatalytic hydrogen production. These results also revealed the potential use of thiolated reduced graphene-oxide and graphitic carbon nitride with silver nanoparticles for enhanced photocatalytic hydrogen production from water.

**Figure 11:**
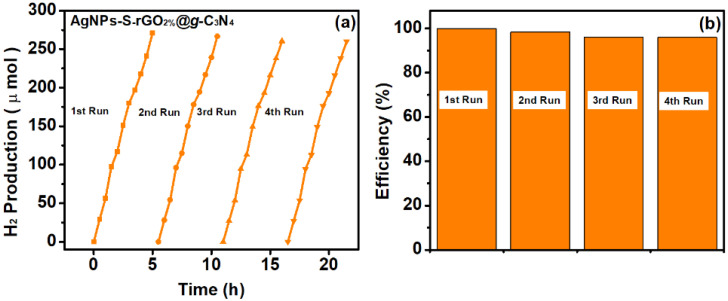
(**a**,**b**) Reusability and stability of AgNPs-S-rGO_2%_@*g*-C_3_N_4_ for four consecutive cycles.

The stability of AgNPs-S-rGO_2%_@*g*-C_3_N_4_ was also investigated by repeating the experiment under the same conditions for four cycles (Figure 11a). The AgNPs-S-rGO_2%_@*g*-C_3_N_4_ was washed multiple times with ethanol and deionised *water* before starting each cycle. The efficiency of the AgNPs-S-rGO_2%_@*g*-C_3_N_4_ (Figure 11b) remained at 96% even after four cycles, supporting the reusability of the synthesised AgNPs-S-rGO_2%_@*g*-C_3_N_4_ catalyst.

**Figure 12:**
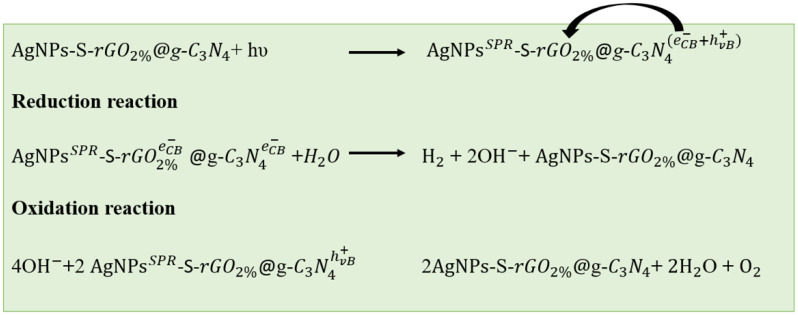
Proposed enhanced hydrogen production mechanism of AgNPs-S-rGO_2%_@*g*-C_3_N_4_.

The photocatalytic production of H_2_, illustrated in Figure 12, elaborates that, in AgNPs-S-rGO_2%_@*g*-C_3_N_4_, the electrons (e^−^) in the VB of *g*-C_3_N_4_ were excited by visible light to the CB, leaving behind holes (h^+^) in the VB. The incorporation of AgNPs in this composite also helped absorb light due to their characteristic SPR and improved the catalytic activity by reducing the recombination of photogenerated electrons and holes. The photoexcited e^−^ in CB of *g*-C_3_N_4_ reacted with water molecules adhered to its surface and generated hydrogen gas. Meanwhile, some of the e^−^ in the CB of the *g*-C_3_N_4_ were transferred to the CB of rGO. Similar to *g*-C_3_N_4_, these electrons (e^−^) in the CB of the rGO also reduced water to H_2_. At the same time, the h^+^ generated in the *g*-C_3_N_4_ oxidised the existing species of hydroxides to water and oxygen molecules.

### 3.6. Photocatalytic CIP Degradation

**Figure 13:**
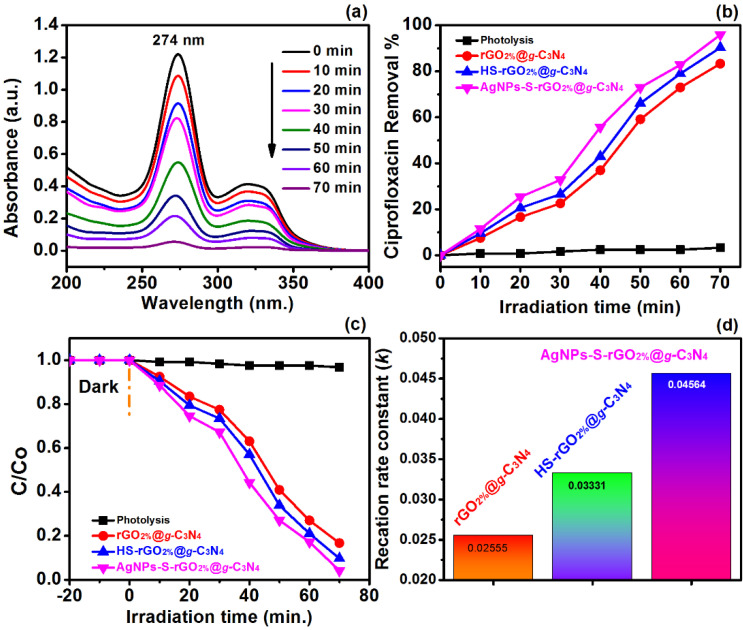
(**a**) UV-Absorption spectra of CIP (**b**) percentage removal of CIP with and without a catalyst (**c**,**d**) reaction rate constant for CIP degradation.

The UV-absorption spectra (Figure 13a) of CIP antibiotics (25 mg/L and pH 7) showed the appearance of the maximum absorption at 274 nm, which is the characterises peak of CIP [57]. This absorption continued down with the increase in the irradiation time. The % removal of CIP with and without a catalyst is shown in Figure 13b. The results revealed 2% removal of CIP in the absence of rGO_2%_@*g*-C_3_N_4_ (photolysis) which also indicated the stability of CIP [58]. However, after adding rGO_2%_@*g*-C_3_N_4,_ its degradation was started and reached 83.29% within 70 min by irradiating under visible light (400 nm, 150 watts). These results well exposed the potential benefit of rGO_2%_@*g*-C_3_N_4_ photocatalyst for the removal of CIP from contaminated water. The CIP degradation was further enhanced to 90.29% and 95.90% with HS-rGO_2%_@*g*-C_3_N_4_ and AgNPs-S-rGO_2%_@*g*-C_3_N_4,_ respectively. The reaction rate was 0.02555, 0.03331 and 0.04564, respectively, for rGO_2%_@*g*-C_3_N_4,_ HS-rGO_2%_@*g*-C_3_N_4_ and AgNPs-S-rGO_2%_@*g*-C_3_N_4_. It is inferred that the reaction rate has reached a maximum value for AgNPs-S-rGO_2%_@*g*-C_3_N_4_ (Figure 13c,d). Generally, the degradation ability by catalytic systems depends on various factors such as its band gap, charge carries recombination ratio etc.

The bandgap of AgNPs-S-rGO_2%_@*g*-C_3_N_4_ was (2.42 eV) which is less in comparison to HS-rGO_2%_@*g*-C_3_N_4_ (2.46 eV) and rGO_2%_@*g*-C_3_N_4_ (2.50 eV). This indicates that the conduction of charge carrier is high for AgNPs-S-rGO_2%_@*g*-C_3_N_4_ in comparison to HS-rGO_2%_@*g*-C_3_N_4_ and rGO_2%_@*g*-C_3_N_4_. Moreover, PL analysis (Figure 3C) also indicated the separation of charge-recombination ratios for AgNPs-S-rGO_2%_@*g*-C_3_N_4_ while moving towards the conduction band. This supports accelerating the conduction of charge carriers, ultimately producing the hydroxyl, reactive oxygen spices and super radical oxides supporting CIP’s enhanced degradation [59].

#### Effect of AgNPs-S-rGO2%@g-C3N4 Dosage over CIP Degradation

**Figure 14:**
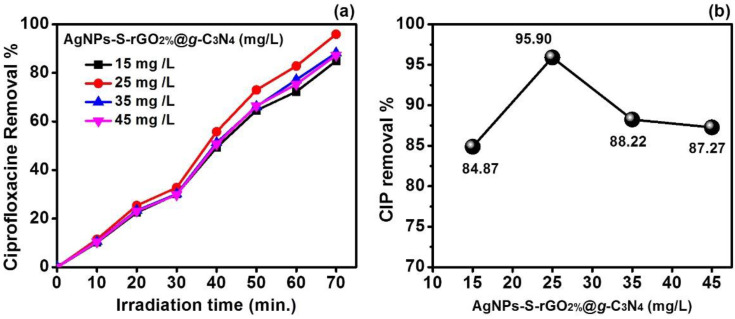
(**a**,**b**) Effect of AgNPs-S-rGO_2%_@*g*-C_3_N_4_ dosage over CIP degradation at constant CIP concentration (25 mg/L) and constant pH-6.

The effect of heterogeneous catalyst dosage may change the reaction rate and efficiency of the photocatalytic process. Therefore, further investigation was carried out at different catalyst dosages of AgNPs-S-rGO_2%_@*g*-C_3_N_4_ while keeping the other reaction parameters such as irradiation time, pH and dye concentrations constant. In this regard, 15 mg/L, 25, 35 and 45 mg/L of AgNPs-S-rGO_2%_@*g*-C_3_N was selected while keeping the CIP concentration fixed, i.e., 25 mg/L and pH was kept constant at 6. The results indicated (Figure 14) the enhanced photocatalytic degradation of CIP, when the dosage of AgNPs-S-rGO_2%_@*g*-C_3_N_4_ increased from 15 mg/L to 25 mg/L. However, after increasing the dosage of AgNPs-S-rGO_2%_@*g*-C_3_N_4_ from 25mg/L, 35, and 45 mg/L, a decline in CIP degradation was noticed. Therefore, maximum CIP degradation was found 84.87%, 95.90%, 88.22% and 87.27%, respectively, for 15 mg/L, 25, 35, and 45 mg/L. These results indicated that the optimal value of AgNPs-S-rGO_2%_@*g*-C_3_N_4_ was 25 mg/L necessary to obtain the maximum degradation of CIP at pH 6. With an increase in the photocatalyst dosage, an obvious increase in the reactive/reaction sites occurred, leading to a high CIP’s photocatalytic degradation rate. This enhanced degradation was attributed to the increase in hydroxyl radical while irradiation. However, a high catalyst dosage of AgNPs-S-rGO_2%_@*g*-C_3_N_4_ beyond its optimal value might lead to suspension opacity, which possibly increased the light scattering inside the reactor and low infiltration of phonons. These factors will eventually decrease the catalytic rate due to the less active catalyst present in the reactor. It can be stated that when present in high concentrations, the catalyst nanoparticles become agglomerated, leading to a decrease in the available reactive active sites. Further deactivation of active catalyst might occur due to the bombardment of activating molecules with molecules at the ground state, which resulted in the decrease of photocatalytic efficiency [60].

## 4. Conclusions

The ratio of rGO to *g*-C_3_N_4_ has a fundamental impact on the photocatalytic CIP degradation and H_2_ production rate. The optimal concentration of rGO will curb the charge recombination ratio of the charged particles at the interface of their heterojunctions. Therefore, the optimum concentration of rGO, i.e., 2 wt.%, in the composite rGO_2%_@*g*-C_3_N_4_, exhibited the maximum H_2_ production rate. Furthermore, the thiolation of rGO and *g*-C_3_N_4_ in HS-rGO_2%_@*g*-C_3_N_4_ also overcame the negative shielding effect of rGO. It decreased the bandgap, building the capability to absorb more photons, which is favourable for enhanced catalytic activity. Moreover, shifting the diffraction peaks for planes of thiolated rGO and *g*-C_3_N_4_ indicated a successful interaction between thiolated functional groups and AgNPs, as confirmed by XPS studies. This interaction also formed smaller AgNPs, keeping the layered assemblies of AgNPs-S-rGO_2%_@*g*-C_3_N_4_ intact, stable and exfoliated. Moreover, C1s and O1s spectra revealed the fundamental role of C=O in the enhanced CIP and hydrogen production activity of AgNPs-S-rGO_2%_@*g*-C_3_N_4_ compared to HS-rGO_2%_@*g*-C_3_N_4_ and rGO_2%_@*g*-C_3_N_4_, which were 3772.5, 1745.2, and 1369.8 μmol g^−1^h^−1^, respectively, under the experimental conditions.

## Figures and Tables

**Table 1:** Ternary composites of *g*-C_3_N_4_/rGO is better than binary composites for photocatalytic hydrogen production.

Binary Photocatalysts	Additive Co-Catalyst	Condition of Sacrificial Agent	Radiation Source	Maximum Hydrogen Yield (µmol h^−1^ g^−1^)	Reference with Year
Thiolated *g*-C_3_N_4_/rGO	Ag	Triethanolamine 15%	300 W Xenon lamp	3772.5	This work
*g*-C_3_N_4_/rGO	MoS_2_	Triethanolamine 0.1%	300 W Xenon lamp	317	[50] 2018
*g*-C_3_N_4_/rGO	MoS2	Sodium sulfite 0.25 Molar	450 Xenon lamp	1650	[54] 2017
*g*-C_3_N_4_/rGO	NiS2	Triethanolamine 1%	300 W Xenon lamp	1555.34	[25] 2019
*g*-C_3_N_4_/rGO	CoMoS2	Triethanolamine 1%	300 W Xenon lamp	684	[55] 2018
*g*-C_3_N_4_/rGO	CdS	Lactic acid 10%	350 W Xenon lamp	1000.5	[56] 2017

## Data Availability

The data presented in this study are available on request from the corresponding author.

## Supplementary Materials

The following supporting information can be downloaded at https://www.mdpi.com/article/10.3390/polym14071290/s1, Figure S1: Comparison of PL spectra for pure *g*-C_3_N_4_, its composites with rGO at various concen-trations of rGO, further its thiolated composites (HS-rGO_2_%@*g*-C_3_N_4_) and AgNPs decorated thio-lated composites (AgNPs-S-rGO_2_%@*g*-C_3_N_4_), Figure S2: XPS survey spectra for rGO_2_%@*g*-C_3_N_4_, HS-rGO_2_%@*g*-C_3_N_4_, and AgNPs-S-rGO_2_%@*g*-C_3_N_4_, Table S1: XPS Elemental composition of AgNPs-S-rGO_2%_@*g*-C_3_N_4_ Survey Spectra.

## References

[1] Zhu J., Zäch M. (2009). Nanostructured materials for photocatalytic hydrogen production. Curr. Opin. Colloid Interface Sci..

[2] Han H.S., Park W., Sivanantham A., Hwang S.W., Surendran S., Sim U., Cho I.S. (2021). Facile fabrication of nanotubular heterostructure for enhanced photoelectrochemical performance. Ceram. Int..

[3] Saraswat S.K., Rodene D.D., Gupta R.B. (2018). Recent advancements in semiconductor materials for photoelectrochemical water splitting for hydrogen production using visible light. Renew. Sustain. Energy Rev..

[4] Chen Y.-C., Huang Y.-S., Huang H., Su P.-J., Perng T.-P., Chen L.-J. (2020). Photocatalytic enhancement of hydrogen production in water splitting under simulated solar light by band gap engineering and localized surface plasmon resonance of ZnxCd1-xS nanowires decorated by Au nanoparticles. Nano Energy.

[5] Han H.S., Park W., Hwang S.W., Kim H., Sim Y., Surendran S., Sim U., Cho I.S. (2020). (020)-Textured tungsten trioxide nanostructure with enhanced photoelectrochemical activity. J. Catal..

[6] Amirav L., Alivisatos A.P. (2010). Photocatalytic Hydrogen Production with Tunable Nanorod Heterostructures. J. Phys. Chem. Lett..

[7] Muthurasu A., Ojha G.P., Lee M., Kim H.Y. (2020). Integration of Cobalt Metal-Organic Frameworks into an Interpenetrated Prussian Blue Analogue to Derive Dual Metal-Organic Framework-Assisted Cobalt Iron Derivatives for Enhancing Electrochemical Total Water Splitting. J. Phys. Chem. C.

[8] Singla S., Sharma S., Basu S., Shetti N.P., Reddy K.R. (2020). Graphene/graphitic carbon nitride-based ternary nanohybrids: Synthesis methods, properties, and applications for photocatalytic hydrogen production. FlatChem.

[9] Bouyarmane H., El Bekkali C., Labrag J., Es-saidi I., Bouhnik O., Abdelmoumen H., Laghzizil A., Nunzi J.M., Robert D. (2021). Photocatalytic degradation of emerging antibiotic pollutants in waters by TiO_2_/Hydroxyapatite nanocomposite materials. Surf. Interfaces.

[10] Ding Q., Lam F.L.Y., Hu X. (2019). Complete degradation of ciprofloxacin over *g*-C_3_N_4_-iron oxide composite via heterogeneous dark Fenton reaction. J. Environ. Manag..

[11] Das K.K., Patnaik S., Mansingh S., Behera A., Mohanty A., Acharya C., Parida K.M. (2020). Enhanced photocatalytic activities of polypyrrole sensitized zinc ferrite/graphitic carbon nitride n-n heterojunction towards ciprofloxacin degradation, hydrogen evolution and antibacterial studies. J. Colloid Interface Sci..

[12] Wang J., Sun Y., Fu L., Sun Z., Ou M., Zhao S., Chen Y., Yu F., Wu Y. (2020). A defective *g*-C_3_N_4_/RGO/TiO_2_ composite from hydrogen treatment for enhanced visible-light photocatalytic H_2_ production. Nanoscale.

[13] Cao S., Fan B., Feng Y., Chen H., Jiang F., Wang X. (2018). Sulfur-doped g-C3N4 nanosheets with carbon vacancies: General synthesis and improved activity for simulated solar-light photocatalytic nitrogen fixation. Chem. Eng. J..

[14] Sun J., Yang S., Liang Z., Liu X., Qiu P., Cui H., Tian J. (2020). Two-dimensional/one-dimensional molybdenum sulfide (MoS_2_) nanoflake/graphitic carbon nitride (*g*-C_3_N_4_) hollow nanotube photocatalyst for enhanced photocatalytic hydrogen production activity. J. Colloid Interface Sci..

[15] Lim Y., Lee D.-K., Kim S.M., Park W., Cho S.Y., Sim U. (2019). Low Dimensional Carbon-Based Catalysts for Efficient Photocatalytic and Photo/Electrochemical Water Splitting Reactions. Materials.

[16] Xu L., Huang W.-Q., Wang L.-L., Tian Z.-A., Hu W., Ma Y., Wang X., Pan A., Huang G.-F. (2015). Insights into Enhanced Visible-Light Photocatalytic Hydrogen Evolution of *g*-C_3_N_4_ and Highly Reduced Graphene Oxide Composite: The Role of Oxygen. Chem. Mater..

[17] Ojha G.P., Pant B., Park S.-J., Park M., Kim H.-Y. (2017). Synthesis and characterization of reduced graphene oxide decorated with CeO_2_-doped MnO_2_ nanorods for supercapacitor applications. J. Colloid Interface Sci..

[18] Jilani A., Rehman G.U., Ansari M.O., Othman M.H.D., Hussain S.Z., Dustgeer M.R., Darwesh R. (2020). Sulfonated polyaniline-encapsulated graphene@graphitic carbon nitride nanocomposites for significantly enhanced photocatalytic degradation of phenol: A mechanistic study. New J. Chem..

[19] Liu G., Niu P., Sun C., Smith S.C., Chen Z., Lu G.Q., Cheng H.-M. (2010). Unique Electronic Structure Induced High Photoreactivity of Sulfur-Doped Graphitic C_3_N_4_. J. Am. Chem. Soc..

[20] Raheman Ar S., Wilson H.M., Momin B.M., Annapure U.S., Jha N. (2020). CdSe quantum dots modified thiol functionalized *g*-C_3_N_4_: Intimate interfacial charge transfer between 0D/2D nanostructure for visible light H_2_ evolution. Renew. Energy.

[21] Jiao J., Wan J., Ma Y., Wang Y. (2016). Facile formation of silver nanoparticles as plasmonic photocatalysts for hydrogen production. RSC Adv..

[22] Gao M., Sun L., Wang Z., Zhao Y. (2013). Controlled synthesis of Ag nanoparticles with different morphologies and their antibacterial properties. Mater. Sci. Eng. C.

[23] Jaiswal A., Pal S., Kumar A., Prakash R. (2020). Metal free triad from red phosphorous, reduced graphene oxide and graphitic carbon nitride (red P-rGO-*g*-C_3_N_4_) as robust electro-catalysts for hydrogen evolution reaction. Electrochim. Acta.

[24] Ibrahim Y.O., Hezam A., Qahtan T.F., Al-Aswad A.H., Gondal M.A., Drmosh Q.A. (2020). Laser-assisted synthesis of Z-scheme TiO_2_/rGO/*g*-C_3_N_4_ nanocomposites for highly enhanced photocatalytic hydrogen evolution. Appl. Surf. Sci..

[25] Pan J., Wang B., Dong Z., Zhao C., Jiang Z., Song C., Wang J., Zheng Y., Li C. (2019). The 2D RGO-NiS_2_ dual co-catalyst synergistic modified *g*-C_3_N_4_ aerogel towards enhanced photocatalytic hydrogen production. Int. J. Hydrogen Energy.

[26] Jilani A., Othman M.H.D., Ansari M.O., Kumar R., Alshahrie A., Ismail A.F., Khan I.U., Sajith V.K., Barakat M.A. (2017). Facile spectroscopic approach to obtain the optoelectronic properties of few-layered graphene oxide thin films and their role in photocatalysis. New J. Chem..

[27] Chua C.K., Pumera M. (2015). Monothiolation and Reduction of Graphene Oxide via One-Pot Synthesis: Hybrid Catalyst for Oxygen Reduction. ACS Nano.

[28] Zhang Y., Pan Q., Chai G., Liang M., Dong G., Zhang Q., Qiu J. (2013). Synthesis and luminescence mechanism of multicolor-emitting *g*-C_3_N_4_ nanopowders by low temperature thermal condensation of melamine. Sci. Rep..

[29] Jilani A., Ansari M.O., Rehman G.u., Shakoor M.B., Hussain S.Z., Othman M.H.D., Ahmad S.R., Dustgeer M.R., Alshahrie A. (2022). Phenol removal and hydrogen production from water: Silver nanoparticles decorated on polyaniline wrapped zinc oxide nanorods. J. Ind. Eng. Chem..

[30] Mudassir M.A., Hussain S.Z., Jilani A., Zhang H., Ansari T.M., Hussain I. (2019). Magnetic Hierarchically Macroporous Emulsion-Templated Poly(acrylic acid)–Iron Oxide Nanocomposite Beads for Water Remediation. Langmuir.

[31] Dai K., Lu L., Liu Q., Zhu G., Wei X., Bai J., Xuan L., Wang H. (2014). Sonication assisted preparation of graphene oxide/graphitic-C3N4 nanosheet hybrid with reinforced photocurrent for photocatalyst applications. Dalton Trans..

[32] Dong F., Wu L., Sun Y., Fu M., Wu Z., Lee S.C. (2011). Efficient synthesis of polymeric *g*-C_3_N_4_ layered materials as novel efficient visible light driven photocatalysts. J. Mater. Chem..

[33] Liu S.-H., Tang W.-T., Chou P.-H. (2020). Microwave-assisted synthesis of triple 2D *g*-C_3_N_4_/Bi_2_WO_6_/rGO composites for ibuprofen photodegradation: Kinetics, mechanism and toxicity evaluation of degradation products. Chem. Eng. J..

[34] Yao X., Zhang W., Huang J., Du Z., Hong X., Chen X., Hu X., Wang X. (2020). Enhanced photocatalytic nitrogen fixation of Ag/B-doped *g*-C_3_N_4_ nanosheets by one-step in-situ decomposition-thermal polymerization method. Appl. Catal. A Gen..

[35] Amendola V. (2016). Surface plasmon resonance of silver and gold nanoparticles in the proximity of graphene studied using the discrete dipole approximation method. Phys. Chem. Chem. Phys..

[36] Cai W., Zhang Y., Jia J., Zhang L. (1998). Semiconducting optical properties of silver/silica mesoporous composite. Appl. Phys. Lett..

[37] Yuan J., Yi X., Tang Y., Liu C., Luo S. (2020). Efficient Photocatalytic Hydrogen Evolution and CO_2_ Reduction: Enhanced Light Absorption, Charge Separation, and Hydrophilicity by Tailoring Terminal and Linker Units in *g*-C_3_N_4_. ACS Appl. Mater. Interfaces.

[38] Hu K., Yao M., Yang Z., Xiao G., Zhu L., Zhang H., Liu R., Zou B., Liu B. (2020). Pressure tuned photoluminescence and band gap in two-dimensional layered *g*-C_3_N_4_: The effect of interlayer interactions. Nanoscale.

[39] Li Y., Zhang H., Liu P., Wang D., Li Y., Zhao H. (2013). Cross-Linked *g*-C_3_N_4_/rGO Nanocomposites with Tunable Band Structure and Enhanced Visible Light Photocatalytic Activity. Small.

[40] Li W., Rodríguez-Castellón E., Bandosz T.J. (2017). Photosensitivity of g-C3N4/S-doped carbon composites: Study of surface stability upon exposure to CO_2_ and/or water in ambient light. J. Mater. Chem. A.

[41] Jo W.-K., Kumar S., Eslava S., Tonda S. (2018). Construction of Bi_2_WO_6_/RGO/*g*-C_3_N_4_ 2D/2D/2D hybrid Z-scheme heterojunctions with large interfacial contact area for efficient charge separation and high-performance photoreduction of CO_2_ and H_2_O into solar fuels. Appl. Catal. B Environ..

[42] Zhang N., Yang M.-Q., Tang Z.-R., Xu Y.-J. (2014). Toward Improving the Graphene–Semiconductor Composite Photoactivity via the Addition of Metal Ions as Generic Interfacial Mediator. ACS Nano.

[43] Yan X., Xu T., Chen G., Yang S., Liu H., Xue Q. (2004). Preparation and characterization of electrochemically deposited carbon nitride films on silicon substrate. J. Phys. D Appl. Phys..

[44] Zheng Y., Yang Y., Zhang Y., Zou W., Luo Y., Dong L., Gao B. (2019). Facile one-step synthesis of graphitic carbon nitride-modified biochar for the removal of reactive red 120 through adsorption and photocatalytic degradation. Biochar.

[45] Cao S., Yu J. (2016). Carbon-based H_2_-production photocatalytic materials. J. Photochem. Photobiol. C Photochem. Rev..

[46] John F., William F., Peter E., Kenneth D. (1995). Handbook of X-ray Photoelectron Spectroscopy.

[47] Das S.K., Dickinson C., Lafir F., Brougham D.F., Marsili E. (2012). Synthesis, characterization and catalytic activity of gold nanoparticles biosynthesized with Rhizopus oryzae protein extract. Green Chem..

[48] Zhang S., Hu L., Wang H., Feng D. (2012). The anti-seizure effect of Ag nanoparticles additive in multialkylated cyclopentanes oil under vacuum condition. Tribol. Int..

[49] Kumar R., Rashid J., Barakat M.A. (2015). Zero valent Ag deposited TiO_2_ for the efficient photocatalysis of methylene blue under UV-C light irradiation. Colloids Interface Sci. Commun..

[50] Yuan Y.-J., Yang Y., Li Z., Chen D., Wu S., Fang G., Bai W., Ding M., Yang L.-X., Cao D.-P. (2018). Promoting Charge Separation in g-C3N4/Graphene/MoS2 Photocatalysts by Two-Dimensional Nanojunction for Enhanced Photocatalytic H_2_ Production. ACS Appl. Energy Mater..

[51] Ong W.-J., Tan L.-L., Chai S.-P., Yong S.-T., Mohamed A.R. (2015). Surface charge modification via protonation of graphitic carbon nitride (*g*-C_3_N_4_) for electrostatic self-assembly construction of 2D/2D reduced graphene oxide (rGO)/*g*-C_3_N_4_ nanostructures toward enhanced photocatalytic reduction of carbon dioxide to methane. Nano Energy.

[52] Lewandowska-Andralojc A., Malolepszy A., Stritt A., Grohmann A. (2020). Modification of eosin Y and cobalt molecular catalyst system with reduced graphene oxide for enhanced photocatalytic hydrogen production. Catal. Sci. Technol..

[53] Das S., Dutta A., Bera R., Patra A. (2019). Ultrafast carrier dynamics in 2D-2D hybrid structures of functionalized GO and CdSe nanoplatelets. Phys. Chem. Chem. Phys..

[54] Wang M., Ju P., Li J., Zhao Y., Han X., Hao Z. (2017). Facile Synthesis of MoS_2_/*g*-C_3_N_4_/GO Ternary Heterojunction with Enhanced Photocatalytic Activity for Water Splitting. ACS Sustain. Chem. Eng..

[55] Xu X., Si Z., Liu L., Wang Z., Chen Z., Ran R., He Y., Weng D. (2018). CoMoS_2_/rGO/C_3_N_4_ ternary heterojunctions catalysts with high photocatalytic activity and stability for hydrogen evolution under visible light irradiation. Appl. Surf. Sci..

[56] Jo W.-K., Selvam N.C.S. (2017). Z-scheme CdS/g-C3N4 composites with RGO as an electron mediator for efficient photocatalytic H_2_ production and pollutant degradation. Chem. Eng. J..

[57] Rogachev A.A., Yarmolenko M.A., Rogachou A.V., Tapalski D.V., Liu X., Gorbachev D.L. (2013). Morphology and structure of antibacterial nanocomposite organic–polymer and metal–polymer coatings deposited from active gas phase. RSC Adv..

[58] Hu K., Li R., Ye C., Wang A., Wei W., Hu D., Qiu R., Yan K. (2020). Facile synthesis of Z-scheme composite of TiO_2_ nanorod/*g*-C_3_N_4_ nanosheet efficient for photocatalytic degradation of ciprofloxacin. J. Clean. Prod..

[59] Akbari S., Moussavi G., Giannakis S. (2021). Efficient photocatalytic degradation of ciprofloxacin under UVA-LED, using S,N-doped MgO nanoparticles: Synthesis, parametrization and mechanistic interpretation. J. Mol. Liq..

[60] Gnanaprakasam A., Sivakumar V.M., Thirumarimurugan M. (2015). Influencing Parameters in the Photocatalytic Degradation of Organic Effluent via Nanometal Oxide Catalyst: A Review. Indian J. Mater. Sci..

