# Supplementary material for: Development and Mechanistic Studies of Ternary Nanocomposites for Hydrogen Production from Water Splitting to Yield Sustainable/Green Energy and Environmental Remediation
Source: Polymers (Basel). 2022 Mar 23;14(7):1290. doi: 10.3390/polym14071290 (PMC9003420; doi:10.3390/polym14071290)
Supplement: Supplementary file 1 [file polymers-14-01290-s001.zip › polymers-1551695-supplementary.pdf]

polymers

MDPI

Supplementary Materials

Development and Mechanistic Studies of Ternary Nanocomposites (AgNPs-S-rGO@*g*-C_3N_4)for Hydrogen Production from Water Splitting to Yield Sustainable/Green Energy and Environmental Remediation

Asim Jilani [1,*], Syed Zajif Hussain [2], Ammar A. Melaibari [1,3] and Nidal H. Abu-Hamdeh [3,4]

1 Center of Nanotechnology, King Abdulaziz University, 21589 Jeddah, Saudi Arabia; aamelaibari@kau.edu.sa
2 Department of Chemistry & Chemical Engineering, SBA-School of Science & Engineering (SBA-SSE), Lahore University of Management Sciences (LUMS), Lahore 54792, Pakistan; syed.hussain@lums.edu.pk
3 Department of Mechanical Engineering, King Abdulaziz University, Jeddah 21589, Saudi Arabia; nabuhamdeh@kau.edu.sa
4 Center of Research Excellence in Renewable Energy and Power system, King Abdulaziz University, Jeddah 21589, Saudi Arabia
* Correspondence: asim.jilane@gmail.com; ajilani@kau.edu.sa, Tel.:+966-599693297

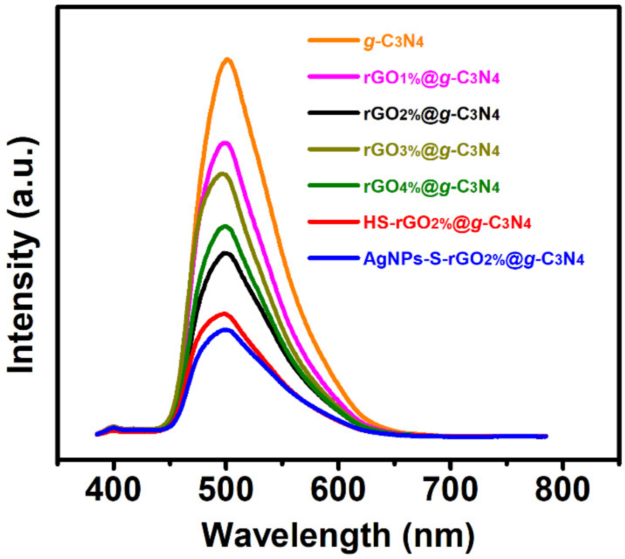

Figure S1. Comparison of PL spectra for pure *g*-C_3N_4, its composites with rGO at various concentrations of rGO, further its thiolated composites (HS-$rGO_{2\%}$@*g*-C_3N_4) and AgNPs decorated thiolated composites (AgNPs-S-$rGO_{2\%}$@*g*-C_3N_4).

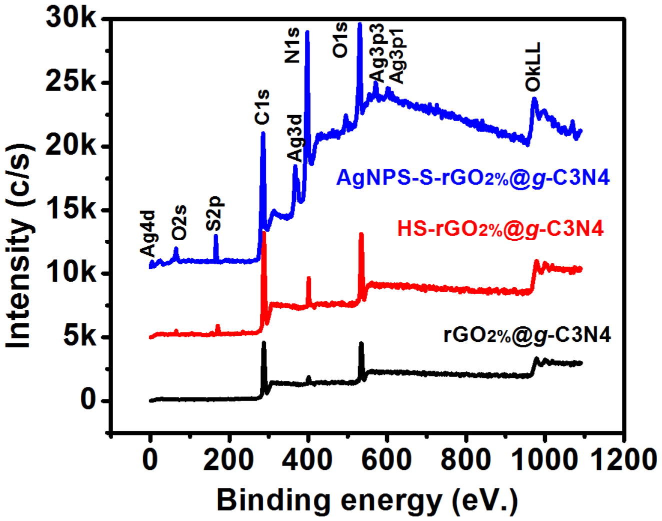

Figure S2. XPS survey spectra for rGO$_{2\%}$@*g*-C_3N_4, HS-rGO$_{2\%}$@*g*-C_3N_4, and AgNPs-S-rGO$_{2\%}$@*g*-C_3N_4.

Table S1. XPS Elemental composition of AgNPs-S-rGO$_{2\%}$@*g*-C_3N_4 Survey Spectra.

Element	Atomic Composition
C1s	49.8
N1s	25.9
O1s	18.3
Ag3P3	1.1
Ag3d3	0.7
S2p	4.2
